# Notoginsenoside R1 Attenuates Oxidative Damage After Intracerebral Hemorrhage by Inhibiting LCN2 and Promoting HO-1 in Astrocytes

**DOI:** 10.3390/brainsci16090958

**Published:** 2026-09-10

**Authors:** Qingyun Liu, Xiao Chen, Baofeng Wang, Yuhao Sun, Liuguan Bian

**Affiliations:** Department of Neurosurgery, Ruijin Hospital, Shanghai Jiao Tong University School of Medicine, Shanghai 200025, China

**Keywords:** intracerebral hemorrhage, oxidative damage, astrocytes, lipocalin-2, Notoginsenoside R1, reactive oxygen species, heme oxygenase-1

## Abstract

Objectives: Intracerebral hemorrhage (ICH) is a devastating form of stroke characterized by high morbidity and mortality, but effective treatment strategy remains in urgent demand. Notoginsenoside R1 (NGR1), a bioactive component extracted from the traditional herb *Panax notoginseng* has been widely applied in the therapy of cardiovascular diseases and neurological disorders. This study aims to investigate the effects of NGR1 on oxidative damage following ICH. Methods: NGR1 was administrated to a collagenase-induced ICH mouse model and hemin-stimulated primary astrocytes in vitro. Hematoma volume, brain water content and neurobehavioral outcomes were assessed in the ICH mice. Cell viability was evaluated using the Cell Counting Kit-8 (CCK-8) assay. In astrocytes pretreated with 25 μM NGR1 for 24 h followed by 30 μM hemin treatment for 24 h, levels of reactive oxygen species (ROS), malondialdehyde (MDA), and the glutathione (GSH)/oxidized glutathione (GSSG) ratio were measured. The expression of lipocalin-2 (LCN2), heme oxygenase-1 (HO-1), and glial fibrillary acidic protein (GFAP) was detected via immunofluorescence staining and Western blotting. Additionally, LCN2 and HO-1 were knocked down in astrocytes, using small interfering RNA (siRNA) transfection. Results: LCN2, HO-1, and GFAP expression was upregulated in the peri-lesional area of the brain following ICH. NGR1 alleviated brain injury and improved neurological function in the collagenase-induced ICH mouse model. Specifically, administration of 20 mg/kg NGR1 suppressed the upregulation of LCN2 and increased HO-1 expression post ICH. NGR1 enhanced cell viability and reversed hemin-induced toxicity in astrocytes in vitro. Treatment with 25 μM NGR1 reduced ROS and MDA levels and upregulated the GSH/GSSG ratio in astrocytes exposed to 30 μM hemin. Furthermore, 25 μM NGR1 inhibited LCN2 and GFAP expression while promoting HO-1 expression in hemin-treated astrocytes. The inhibitory effect of NGR1 on LCN2 was reversed by the Nrf-2/HO-1 inhibitor ML385. Moreover, LCN2 knockdown promoted HO-1 expression and suppressed ROS levels in astrocytes. Conversely, HO-1 knockdown increased LCN2 and GFAP expression, thereby reversing the protective effects of NGR1. Conclusions: This study demonstrates that NGR1 alleviates ICH-induced oxidative damage by promoting HO-1 expression and inhibiting LCN2 expression, thereby maintaining redox homeostasis.

## 1. Introduction

Intracerebral hemorrhage (ICH) is one of the major causes of death and disability around the world [1]. The global incidence of ICH is 43.65 per 100,000 person-years, a figure that is markedly increasing due to an aging population and the widespread use of antithrombotic agents [2,3]. Consequently, there is an urgent need for effective therapeutic strategies for ICH [4].

ICH is typically associated with chronic hypertension and cerebral amyloid angiopathy, which contribute to blood vessel rupture and subsequent leakage of blood into the brain parenchyma [5]. Primary injury results from physical damage to adjacent areas due to the mass effect and expansion of the hematoma [6]. Secondary injury is driven by toxic factors released from injured tissue and erythrocyte lysis, inducing oxidative damage and neuroinflammation in the perihematomal region [7]. Following ICH, hemoglobin released from red blood cells and its degradation products, such as heme and iron, lead to the generation of reactive oxygen species (ROS), contributing to cell toxicity and promoting neuroinflammation [8]. During the secondary phase of ICH, heme oxygenase-1 (HO-1) plays a crucial role in promoting heme degradation and reducing ROS generation [9]. Furthermore, previous research has shown that selective overexpression of HO-1 in the astrocytes of ICH mice is associated with reduced mortality and improved neurological outcomes [10]. Therefore, upregulating HO-1 represents a promising approach to attenuating oxidative damage after ICH.

Astrocytes are the most prevalent cells in the human brain and play a pivotal role in maintaining brain homeostasis [11]. Astrocytes exhibit antioxidant capabilities that protect neurons against oxidative damage [12]. However, in the presence of cerebral insults such as ICH, astrocytes are stimulated and transform into reactive astrocytes [13]. Although reactive astrocytes exhibit heterogeneity, they have traditionally been classified into binary phenotypes: A1 (neurotoxic) and A2 (neuroprotective) [14]. However, recent evidence suggests that molecular expression and functional changes in reactive astrocytes are plastic and can change over time without altering the cell subtype; thus, referring to differences in “reactive astrocyte states” rather than distinct subtypes is more appropriate [15]. Reactive astrocytes generate hydrogen peroxide, leading to neuronal loss and cognitive impairment through oxidative damage [16]. Additionally, chemokines produced by reactive astrocytes exacerbate neuroinflammation [17]. Lipocalin-2 (LCN2), a member of the lipocalin superfamily, has been implicated in neuroinflammation [18]. Recent studies indicate that LCN2 is significantly upregulated in the brain following injury, whereas it is barely detectable in healthy brains [19,20]. Astrocytes are the primary producers of LCN2 in the brain. LCN2 drives the activation of reactive astrocytes toward an inflammatory phenotype, contributing to enhanced neuroinflammation and neuronal death [21,22]. Notably, co-localization of LCN2 expression with microglia or neurons was not detected after traumatic brain injury [23]. LCN2 modulates astrocyte activation and polarization through various signaling pathways, such as NF-κB and JAK-STAT, amplifying the inflammatory response and aggravating brain injury [24]. Moreover, in vivo knockdown of LCN2 ameliorates brain injury in ICH mice [25]. It has also been reported that LCN2 mediates ROS generation [26]. As a crucial regulatory molecule in neuroinflammation, LCN2 mediates multiple signaling networks and is considered to have significant therapeutic potential [24]. Therefore, we hypothesize that inhibiting LCN2 in astrocytes prevents their polarization into pro-inflammatory reactive states, thereby alleviating brain injury after ICH.

Notoginsenoside R1 (NGR1) is a bioactive component extracted from *Panax notoginseng* and is widely used in the treatment of cardiovascular and neurological disorders [27]. Previous studies have demonstrated that NGR1 exhibits antioxidant, anti-inflammatory, anti-apoptotic, and neuroprotective properties [28]. Numerous reports indicate that NGR1 prevents oxidative damage by promoting HO-1 expression and suppressing ROS generation in various diseases [29,30]. Consequently, we postulate that NGR1 alleviates oxidative damage after ICH by promoting HO-1 expression and inhibiting ROS generation.

To investigate the therapeutic effect of NGR1 on oxidative damage following ICH, we applied NGR1 treatment to both in vivo and in vitro ICH models. To elucidate the underlying mechanisms, we detected LCN2 and HO-1 expression levels as well as redox states. This study demonstrates that NGR1 inhibits LCN2 expression and promotes HO-1 expression and the GSH/GSSG ratio, thereby suppressing ROS generation and MDA levels in astrocytes and alleviating oxidative damage after ICH. This research identifies a potential drug that attenuates brain injury by inhibiting the pro-inflammatory reactive astrocyte polarization modulator LCN2 and maintaining redox homeostasis via HO-1 promotion, thus providing a feasible therapeutic strategy for ICH.

## 2. Materials and Methods

### 2.1. Animals

Male C57BL/6 mice (8 weeks old, 25 g) were obtained from Zhejiang Vital River Laboratories (Jiaxing, China). All experiments were conducted in accordance with the guidelines of the Institutional Animal Care and Use Committee of Shanghai Jiao Tong University. Mice were housed in a controlled environment with a 12 h light/dark cycle and free access to food and water.

### 2.2. Animal Model of ICH

Adult C57BL/6 mice were anesthetized with 1.5% isoflurane and secured in a stereotactic frame (RWD Life Science, Shenzhen, China). ICH was induced by collagenase IV injection as previously described [31,32]. Briefly, 0.075 U of collagenase IV dissolved in 0.4 μL of PBS was infused over 4 min into the striatum (3 mm deep, 0.5 mm anterior, and 2.2 mm lateral to the bregma. As schematic diagram shows in Figure S1A). The sham group received an equivalent volume of PBS.

### 2.3. Brain Water Content

Three days post-surgery, mice were sacrificed, and brains were immediately isolated. Brain tissues were weighed to determine wet weight, then dried at 100–105 °C for 72 h to determine dry weight. Brain water content was calculated as (wet weight − dry weight)/wet weight × 100%.

### 2.4. Brain Hematoma Volume

Three days post-surgery, mice were deeply anesthetized and perfused with PBS to collect the brains. Coronal brain sections (1 mm thick) were prepared using a mouse brain matrix (RWD Life Science, Shenzhen, China), and digital images were captured. Hematoma areas in each section were measured using ImageJ software 13.0.6 (NIH, Maryland, MD, USA). Hematoma volume was calculated as the sum of the hematoma areas multiplied by the section thickness.

### 2.5. Neurobehavioral Tests

Behavioral assessments, including modified neurological severity scores (mNSS), rotarod test, and grid walking test, were performed at 1, 3, 7, and 14 days post-ICH. All evaluations were conducted by an experimenter blinded to the treatment groups.

mNSS. The mNSS comprises motor tests (0–6 points), sensory tests (0–2 points), beam balance tests (0–6 points), and reflexes/abnormal movements (0–4 points). Scores range from 0 to 18, with 0 indicating normal function and 18 indicating severe impairment.

Rotarod test. This test evaluates balance, grip strength, and sensorimotor coordination. Mice underwent training for 3 consecutive days prior to surgery. The rod speed increased from 20 to 40 rpm over 5 min. Post-surgery, mice were tested at 35 rpm for 5 min at 3, 7, and 14 days. Each mouse was tested three times, and the latency to fall was recorded.

Grid walking test. This test assesses descending motor control impairment. Mice were placed in the center of a wire grid platform (32 cm × 20 cm × 50 cm) with 1 cm openings. Each mouse explored the platform for 5 min while being recorded by a camera positioned underneath. Videos were analyzed to record total steps and foot faults (instances where paws slipped through the grid). The foot fault index was calculated as [(contralateral faults − ipsilateral faults)/total steps] × 100.

### 2.6. NGR1 Administration

Mice were randomly divided into five groups to determine the optimal dose for ICH treatment: (1) sham (n = 15), (2) ICH (n = 15), (3) ICH + 10 mg/kg NGR1 (n = 15), (4) ICH + 20 mg/kg NGR1 (n = 15), and (5) ICH + 40 mg/kg NGR1 (n = 15). After confirming the effective dose, mice were randomly divided into three groups for subsequent experiments: (1) sham (n = 20), (2) ICH (n = 20), and (3) ICH + 20 mg/kg NGR1 (n = 20). Following collagenase infusion, NGR1 (Winherb, Shanghai, China) dissolved in PBS via ultrasonication was immediately administered via intraperitoneal (i.p.) injection, while the sham and ICH groups received an equivalent volume of PBS. NGR1 (20 mg/kg) was administered every 24 h from the day of surgery until day 3 post-ICH. The number of mice per experiment was as follows: brain water content (n = 5/group, total n = 25); hematoma volume analysis (n = 5/group, total n = 25); neurobehavioral tests (n = 10/group, total n = 30); immunofluorescence (IF) (n = 5/group, total n = 25); Western blot (n = 3–5/group, total n = 30).

### 2.7. Tissue Collection

Mice were deeply anesthetized and perfused with cold PBS followed by 4% paraformaldehyde (PFA) at 1 and 3 days post-operation. Brains were post-fixed for 12 h, dehydrated in 30% sucrose for 48 h, and stored at –80 °C for cryosectioning. Brain cryosections (20 μm) were collected for immunostaining. For Western blotting, mice were perfused with cold PBS, and peri-lesional brain slices (2 mm anterior and posterior to the injection site) were collected using a mouse brain matrix.

### 2.8. Primary Astrocytes Isolation and Culture

Primary astrocytes were isolated from the cortex of newborn C57BL/6 mice (JSJ, Shanghai, China) as previously reported [33]. Cells were suspended in DMEM supplemented with 10% FBS and 1% penicillin–streptomycin (P/S) and plated on poly-D-lysine-coated T75 flasks (Sigma, St. Louis, MO, USA). Cells were maintained at 37 °C, 95% humidity, and 5% CO_2_. The medium was changed 24 h after seeding and every 3 days thereafter. Upon confluence, microglia and oligodendrocyte precursor cells were removed by shaking on an orbital shaker at 220 rpm for 6 h to obtain purified astrocytes for subsequent experiments.

### 2.9. Cell Viability Measurement

The effects of hemin or NGR1 on astrocytes were assessed using the Cell Counting Kit-8 (CCK-8, Beyotime, Nantong, China, C0037), according to the manufacturer’s protocol. Astrocytes were plated in 96-well plates. One day after seeding, cells were treated with or without NGR1 for 24 h, followed by hemin treatment for 24 h. The medium was replaced with 100 μL DMEM containing 10 μL CCK-8. After incubation for 1 h at 37 °C with 5% CO_2_, absorbance at 450 nm was measured using a microplate reader (SpectraMax, Sunnyvale, CA, USA). Cell viability was expressed as a percentage of the control group.

### 2.10. Drug Treatment of Astrocytes

Hemin chloride (Santa Cruz Biotechnology, Santa Cruz, CA, USA, sc-202646) was dissolved in NaOH and diluted with PBS. NGR1 and the Nrf-2/HO-1 inhibitor ML385 (TargetMol, Boston, MA, USA, T4360) were dissolved in DMSO and diluted with DMEM. Astrocytes were plated in 6 cm dishes and treated with or without 25 μM NGR1 for 24 h, followed by 30 μM hemin treatment for 24 h. To inhibit HO-1 expression, 10 μM ML385 was administered 2 h prior to NGR1 treatment.

### 2.11. Small Interfering RNA (siRNA) Transfection

Astrocytes were transfected with 50 nM siRNA or negative control siRNA (si-NC) using Lipofectamine™ RNAiMAX (Invitrogen, Carlsbad, CA, USA, #13778-030) for 24 h, according to the manufacturer’s instructions. Knockdown efficiency was evaluated by Western blot. The sequences were as follows: si-LCN2 (OBiO, Shanghai, China): 5′-CAAGAGAACAAUAGCUACATT-3′; si-HO-1 (Tsingke Biotech, Beijing, China): 5′-GCCACACAGCACUAUGUAAdTdT-3′; si-NC (SYNBIO, Suzhou, China): 5′-UUCUCCGAACGUGUCACGUTT-3′.

### 2.12. ROS Detection

ROS levels in astrocytes were detected using an ROS Assay Kit (Beyotime, Nantong, China, S0033). Astrocytes were seeded in 6-well plates. After drug treatment, the medium was replaced with DMEM containing 10 μM DCFH-DA, and cells were incubated at 37 °C for 20 min. Cells were washed three times with PBS, and ROS was visualized by DCF fluorescence under a fluorescence microscope. Images of three fields per coverslip were acquired, and the number of ROS-positive cells was counted.

### 2.13. MDA and GSH/GSSH Measurement

To assess redox states in astrocytes, MDA and GSH/GSSG levels were measured. MDA was detected using an MDA Assay Kit (Beyotime, Nantong, China, S0131S), according to the manufacturer’s protocol. GSH and GSSG levels were measured using a GSH and GSSG Assay Kit (Beyotime, Nantong, China, S0053), and the GSH/GSSG ratio was calculated.

### 2.14. Immunofluorescence Staining

Brain cryosections or astrocyte coverslips fixed with 4% PFA were rinsed three times with PBS for 5 min, permeabilized with 0.3% Triton X-100 for 10 min, and blocked with 10% FBS for 1 h after being washed 3 times with PBS. Sections or coverslips were incubated with primary antibodies overnight at 4 °C, followed by appropriate secondary antibodies at 37 °C for 60 min. Nuclei were counterstained with DAPI. Fluorescence images were captured at 1024 × 1024 resolution under 40× magnification using a confocal laser-scanning system (Leica, Solms, Germany). The peri-lesional area was defined as the region surrounding the hematoma. Three non-overlapping fields in coronal slices (Figure S1B) were randomly selected for imaging and quantification by a researcher blinded to group assignment. Primary antibodies used were: goat anti-LCN2 polyclonal (R&D, Minneapolis, MN, USA, AF1857, 1:200); rabbit anti-GFAP polyclonal (Servicebio, Wuhan, China, GB11096, 1:500); rabbit anti-HO-1 polyclonal (Abcam, Cambridge, UK, ab13243, 1:500); and mouse anti-Nrf-2 monoclonal (ProteinTech, Wuhan, China, 66504-1-Ig, IF 1:200, WB 1:3000).

### 2.15. Western Blot

Whole-cell or brain proteins were extracted using RIPA lysis buffer supplemented with PMSF, protease cocktail inhibitor, and phosphatase inhibitor. Equal amounts of protein (30 μg) were loaded onto 10% SDS-PAGE gels and transferred onto PVDF membranes (Millipore, Billerica, MA, USA). Membranes were blocked with protein-free rapid blocking buffer (Epizyme, Shanghai, China) and incubated with primary antibodies overnight, followed by appropriate secondary antibodies at room temperature for 1 h. After three 5 min washes, membranes were incubated with ECL solution, and images were acquired using an e-blot touch imager (e-Blot, Shanghai, China). The primary antibodies used were: goat anti-LCN2 polyclonal (R&D, Minneapolis, MN, USA, AF1857, 1:800); rabbit anti-HO-1 polyclonal (Abcam, Cambridge, UK, ab13243, 1:3000); mouse anti-GFAP monoclonal (Servicebio, Wuhan, China, GB12096, 1:2000); and mouse anti-GAPDH monoclonal (Servicebio, Wuhan, China, GB12002, 1:1000).

### 2.16. Statistical Analyses

All values are presented as mean ± standard deviation (SD). Statistical analyses were performed using GraphPad Prism 8.0 (GraphPad, San Diego, CA, USA). Data normality was checked, and analyses were conducted using one-way analysis of variance (ANOVA) with Dunnett’s test. Statistical significance was set at *p* < 0.05.

## 3. Results

### 3.1. LCN2, HO-1, and GFAP Expression Were Upregulated in the Collagenase-Induced ICH Mouse Model

**Figure 1:**
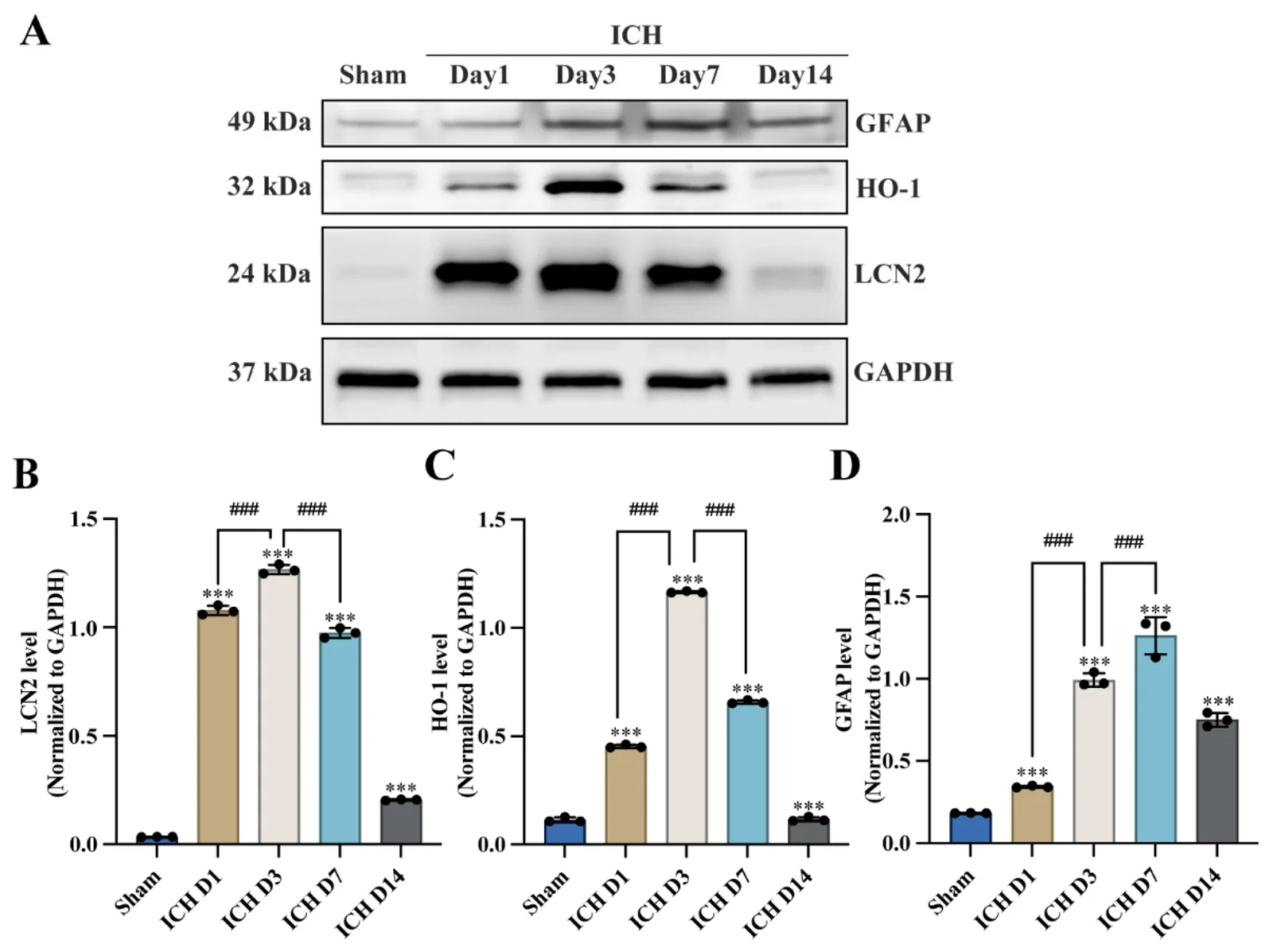
LCN2, HO-1 and GFAP expression were promoted by collagenase-induced ICH mouse model. (**A**) Lipocalin-2 (LCN2), heme oxygenase-1 (HO-1) and GFAP levels in peri-lesion area of the mouse brain tissue at day 1, 3, 7, 14 after intracerebral hemorrhage (ICH). (**B**–**D**) Quantification of LCN2 (**B**), HO-1 (**C**) and GFAP (**D**) protein levels (n = 3 mice per group). One-way ANOVA followed by Dunnett’s test. *** *p* < 0.001 vs. sham group; ### *p* < 0.001, vs. day 3 of ICH (ICH D3). All data are presented as mean ± SD.

LCN2 has been reported to be upregulated in the brain when insults are encountered and is related to pro-inflammation astrocytes phenotype-mediated oxidative damage. To investigate the change in astrocyte expression after ICH, Western blot was performed after ICH. There was a significant increase in the LCN2 protein from day 1 after ICH and maintained at a high level to day 7 after ICH (Figure 1A,B). Moreover, HO-1 was obviously induced by ICH, especially on day 3 after ICH (Figure 1A,C). GFAP was also stimulated by ICH and significantly upregulated on day 7 of ICH (Figure 1A,C).

### 3.2. NGR1 Alleviated Brain Injury and Improved Neurofunction in Collagenase-Induced ICH Mouse Model

**Figure 2:**
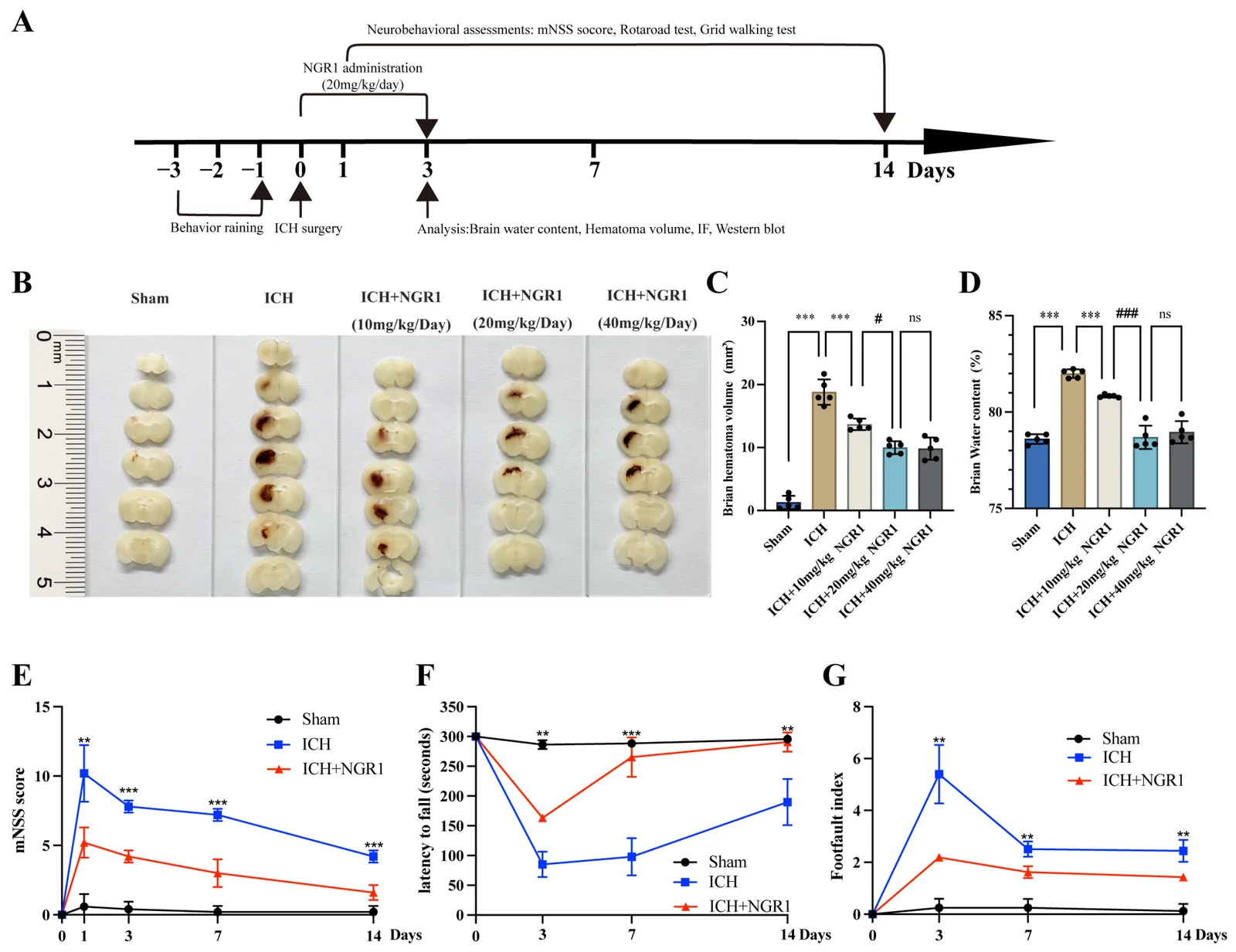
NGR1 alleviated brain injury and improved neurofunction in collagenase-induced ICH mouse model. (**A**) Schematic treatment timeline of the experimental design. NGR1, notoginsenoside R1; IF, immunofluorescent staining; mNSS, modified neurologic severity score. (**B**) Representative coronal sections of brain from mouse treated with NGR1 consecutively for 3 days after ICH. (**C**) The volume quantification of hematoma in NGR1-treated mice at day 3 (n = 5). One-way ANOVA followed by Dunnett’s test. *** *p* < 0.001 vs. ICH group; ns means no significance, # *p* < 0.05 vs. ICH + 20 mg/kg group. (**D**) Brain water content at 3 days post-surgery (n = 5). One-way ANOVA followed by Dunnett’s test. *** *p* < 0.001 vs. ICH group; ns means no significance, ### *p* < 0.001, vs. ICH + 20 mg/kg group. (**E**–**G**) Neurobehavioral results were evaluated by neurobehavioral tests including the mNSS (**E**), rotarod test (**F**), and grid walking test (**G**). n = 10 mice per group. Two-way ANOVA followed by Bonferroni multiple comparisons test ** *p* < 0.01, *** *p* < 0.001 compared with the ICH group. All data are presented as mean ± SD.

To investigate the effect of NGR1 on ICH-induced brain injury, NGR1 was administered to the collagenase-induced ICH mouse model. NGR1 reduced hematoma volume. Specifically, administration of 20 mg/kg NGR1 for 3 days post-ICH significantly diminished hematoma volume more effectively than the 10 mg/kg dose, while yielding effects comparable to those of the 40 mg/kg dose (Figure 2B,C). Brain water content was measured to evaluate brain edema. NGR1 alleviated ICH-induced edema, with the 20 mg/kg dose significantly reducing water content compared to the 10 mg/kg dose and exhibiting effects comparable to those of the 40 mg/kg dose (Figure 2D). Although no significant difference in protective efficacy was observed between the 20 mg/kg and 40 mg/kg doses, the 20 mg/kg dose was selected for subsequent experiments due to its lower dosage. Neurological function was evaluated using mNSS, rotarod, and grid walking tests. Compared to the ICH group, 20 mg/kg NGR1 administration significantly decreased mNSS scores (Figure 2E), increased latency to fall on the rotarod (Figure 2F), and reduced foot faults in the grid walking test (Figure 2G). Thus, NGR1 alleviated brain injury by reducing hematoma volume and brain water content, leading to improved neurological function.

### 3.3. NGR1 Administration Suppressed LCN2 Expression and Promoted HO-1 Expression in ICH Mouse Model

**Figure 3:**
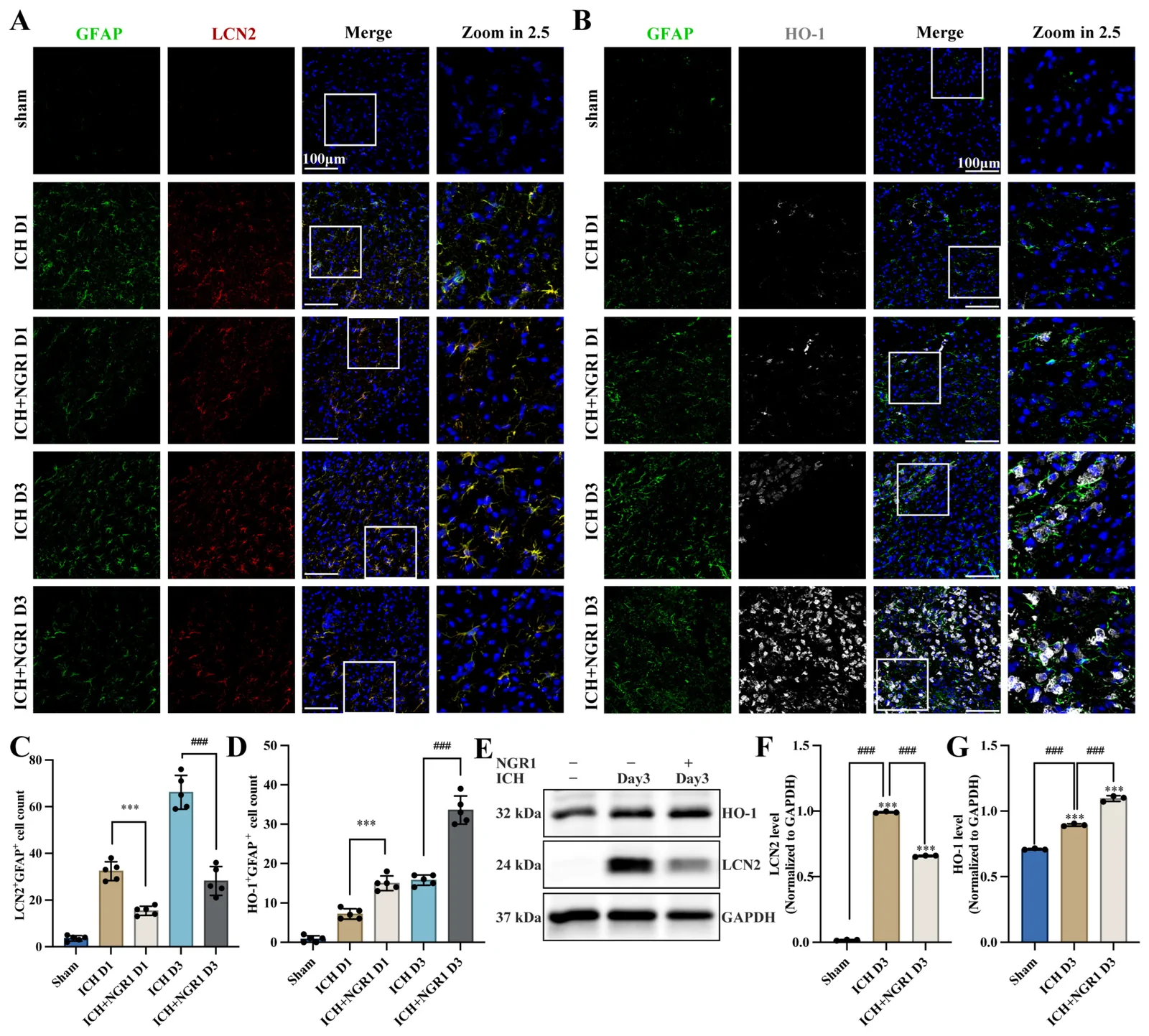
**NGR1 administration suppressed LCN2 expression and promoted HO-1 expression in ICH mouse model.** (**A**,**B**) Representative immunofluorescence staining images of astrocytes (GFAP, green) co-localized with LCN2 (**A**), HO-1 (**B**) in the peri-lesion area of mice treated with 20 mg/kg NGR1 at day 1 and 3 after ICH. Scale bar = 100 μm. (**C**,**D**) The number of GFAP^+^LCN2^+^ cells (**C**) and GFAP^+^HO-1^+^ cells (**D**). n = 5 mice per group. One-way ANOVA followed by Dunnett’s test. *** *p* < 0.001 vs. ICH D1 group; ### *p* < 0.001, vs. ICH day 3 group. (**E**) Representative immunoblots of LCN2 and HO-1protein levels in peri-lesion area of NGR1 treated mice at day 3. (**F**,**G**). Quantification of LCN2 (**F**) and HO-1 (**G**) protein levels (n = 3 mice per group). One-way ANOVA followed by Dunnett’s test. *** *p* < 0.001 vs. sham group; ### *p* < 0.001, vs. ICH D3 group. All data are presented as mean ± SD.

To explore the mechanism of NGR1’s protective effect against ICH-mediated brain injury, immunofluorescence staining and Western blotting were performed. There was a significant increase in LCN2-positive cells in the peri-hematoma area, which largely co-localized with GFAP-positive cells. However, administration of 20 mg/kg NGR1 significantly decreased the number of LCN2^+^/GFAP^+^ cells compared to the ICH group (Figure 3A,C). Additionally, HO-1-positive cells were upregulated after ICH, and NGR1 treatment significantly increased the number of HO-1-positive cells around the hematoma at 3 days post-ICH (Figure 3B,D). Western blot analysis confirmed these findings: LCN2 expression was significantly upregulated at 3 days post-ICH, and NGR1 markedly suppressed LCN2 expression (Figure 3E,F). Moreover, NGR1 significantly promoted HO-1 expression compared to the ICH group (Figure 3E,G).

### 3.4. NGR1 Reversed Cell Toxicity Induced by Hemin in Astrocytes

**Figure 4:**
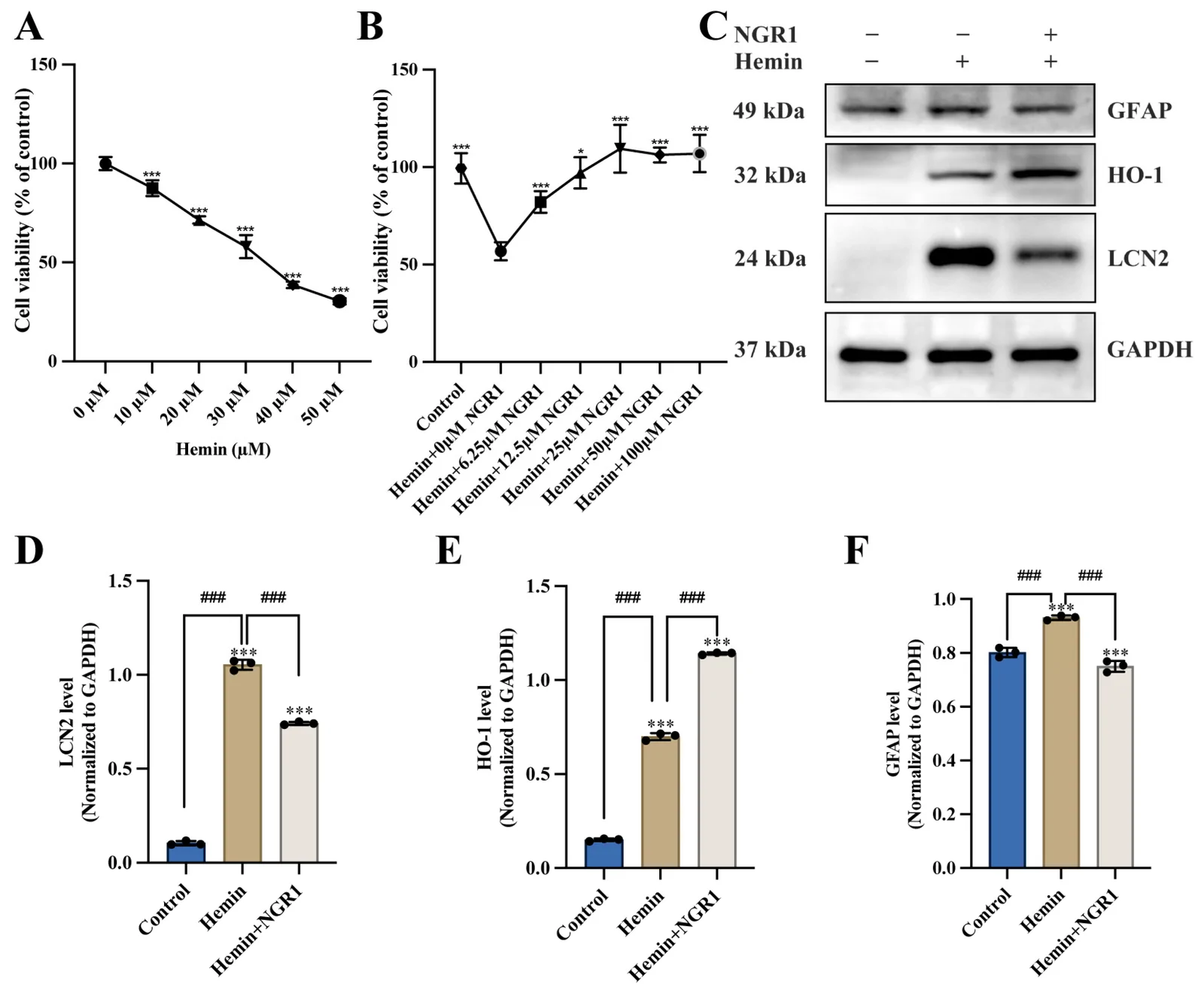
NGR1 elevated cell viability, promoted HO-1 level and inhibited LCN2 and GFAP level in hemin-treated astrocytes. (**A**,**B**) Astrocytes were exposed to a hemin range from 0 to 50 μM (**A**) or an NGR1 range from 0 to 100 μM for 24 h followed by 30 μM hemin treatment 24 h (**B**); then cell viability was evaluated by CCK-8. n = 3 biologically independent astrocyte cultures. One-way ANOVA followed by Dunnett’s test. * *p* < 0.05, *** *p* < 0.001 vs. control group. (**C**) Representative immunoblots of LCN2, HO-1 and GFAP levels in astrocytes pre-incubated with 25 μM NGR1 for 24 h followed by 30 μM hemin treatment for 24 h. (**D**,**F**) Quantification of LCN2 (**D**), HO-1 (**E**) and GFAP (**F**) protein levels. n = 3 biologically independent astrocyte cultures. One-way ANOVA followed by Dunnett’s test. *** *p* < 0.001 vs. control group; ### *p* < 0.001, vs. hemin group. All data are presented as mean ± SD.

Astrocytes play a critical role in neuroinflammation and oxidative injury. To determine whether the protective effect of NGR1 is mediated by astrocytes, in vitro experiments were conducted. Hemin, derived from erythrocytes, is a key factor in secondary brain damage after ICH and was used to model ICH in vitro. Cell viability was assessed to determine the appropriate doses of hemin and NGR1. Hemin impaired cell viability, with 30 μM hemin significantly reducing viability by 50% which was selected for subsequent experiments (Figure 4A). NGR1 enhanced cell viability, with 25 μM NGR1 reversing hemin-induced toxicity in astrocytes (Figure 4B).

### 3.5. NGR1 Treatment Promoted HO-1 Expression and Inhibited LCN2 and GFAP Expression in Hemin-Treated Astrocytes

**Figure 5:**
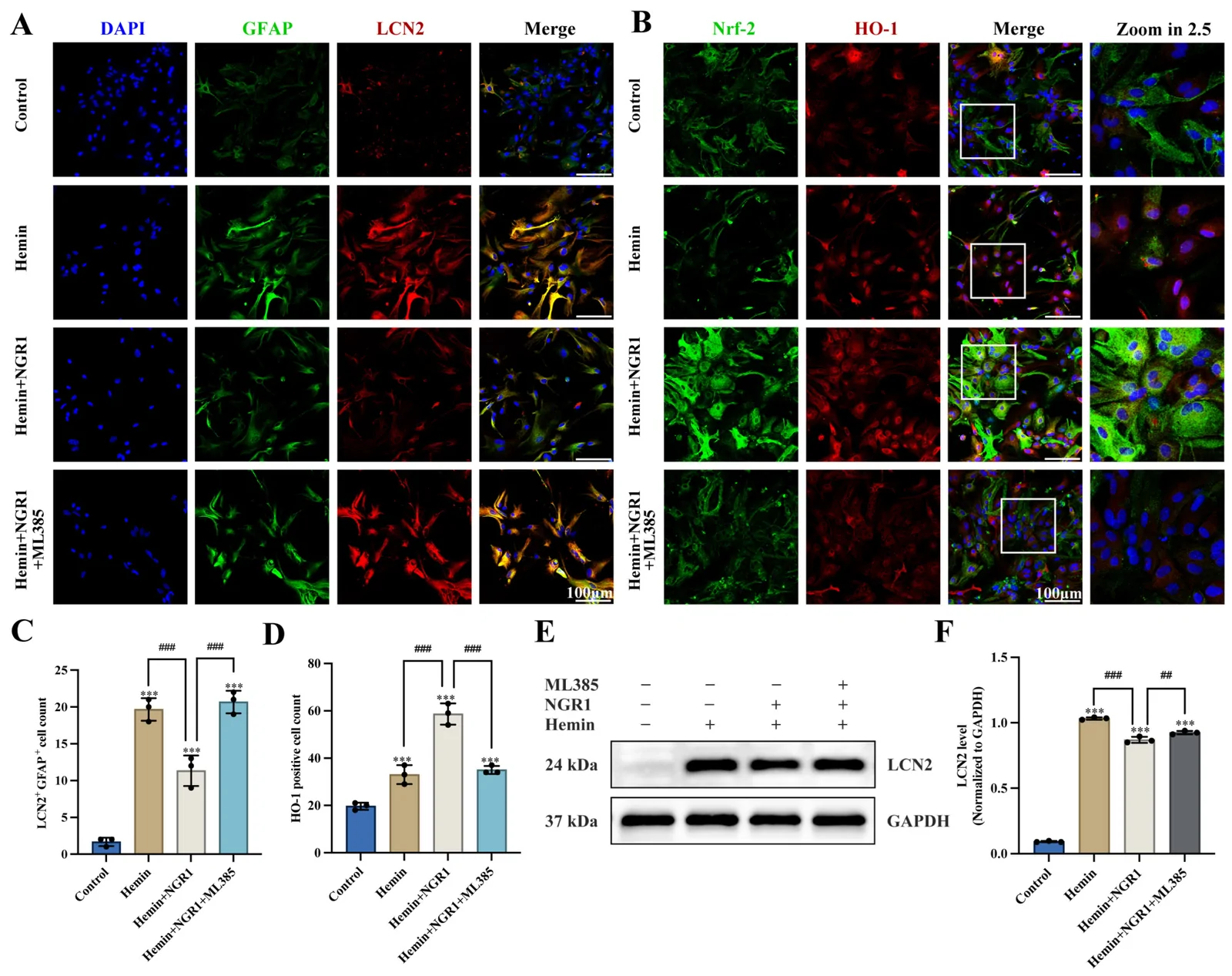
NGR1 treatment suppressed LCN2 expression and promoted HO-1 expression in in hemin-treated astrocytes (**A**,**B**) Representative immunofluorescence staining images of GFAP co-staining with LCN2 (**A**), HO-1 co-staining with Nrf-2 (**B**) in astrocytes incubated with 25 μM NGR1 for 24 h followed by 30 μM hemin treatment for 24 h, astrocytes were treated with 10 μM Nrf-2/HO-1 inhibitor ML385 for 2 h before NGR1 incubation in hemin + NGR1 + ML385 group. (**C**,**D**) The number of GFAP^+^LCN2^+^ cells (**C**) and HO-1^+^ cells (**D**). n = 3 biologically independent astrocyte cultures. One-way ANOVA followed by Dunnett’s test. *** *p* < 0.001 vs. control group; ### *p* < 0.001, vs. hemin + NGR1 group. (**E**) Representative immunoblots of LCN2 in astrocytes incubated with 25 μM NGR1 for 24 h followed by 30 μM hemin treatment for 24 h; in hemin + NGR1 + ML385 group, astrocytes were treated with 10μM Nrf-2/HO-1 inhibitor ML385 for 2 h before NGR1 incubation. (**F**) Quantification of LCN2 protein levels. N = 3 biologically independent astrocyte cultures. One-way ANOVA followed by Dunnett’s test. *** *p* < 0.001 vs. control group; ## *p* < 0.01, ### *p* < 0.001, vs. hemin + NGR1 group. All data are presented as mean ± SD.

To investigate the effect of NGR1 on oxidative damage, astrocytes were pretreated with 25 μM NGR1. Western blot analysis revealed that 25 μM NGR1 visibly promoted HO-1 protein levels (Figure 4C,D) and significantly inhibited LCN2 and GFAP protein levels (Figure 4C,E,F). Similarly, NGR1 treatment significantly reduced the number of LCN2^+^GFAP^+^ cells induced by hemin, which was reversed by 10 μM ML385 (Figure 5A,C). Moreover, NGR1 remarkably increased the number of HO-1 positive cells, which was blocked by 10 μM ML385 as well (Figure 5B,D). Furthermore, Western blot analysis showed that ML385 treatment significantly impaired the suppressive effect of NGR1 on LCN2 (Figure 5E,F).

### 3.6. NGR1 Ameliorated Oxidative Injury in Hemin-Treated Astrocytes by Suppressing ROS Level and MDA Level, Raising the GSH/GSSH Ratio

**Figure 6:**
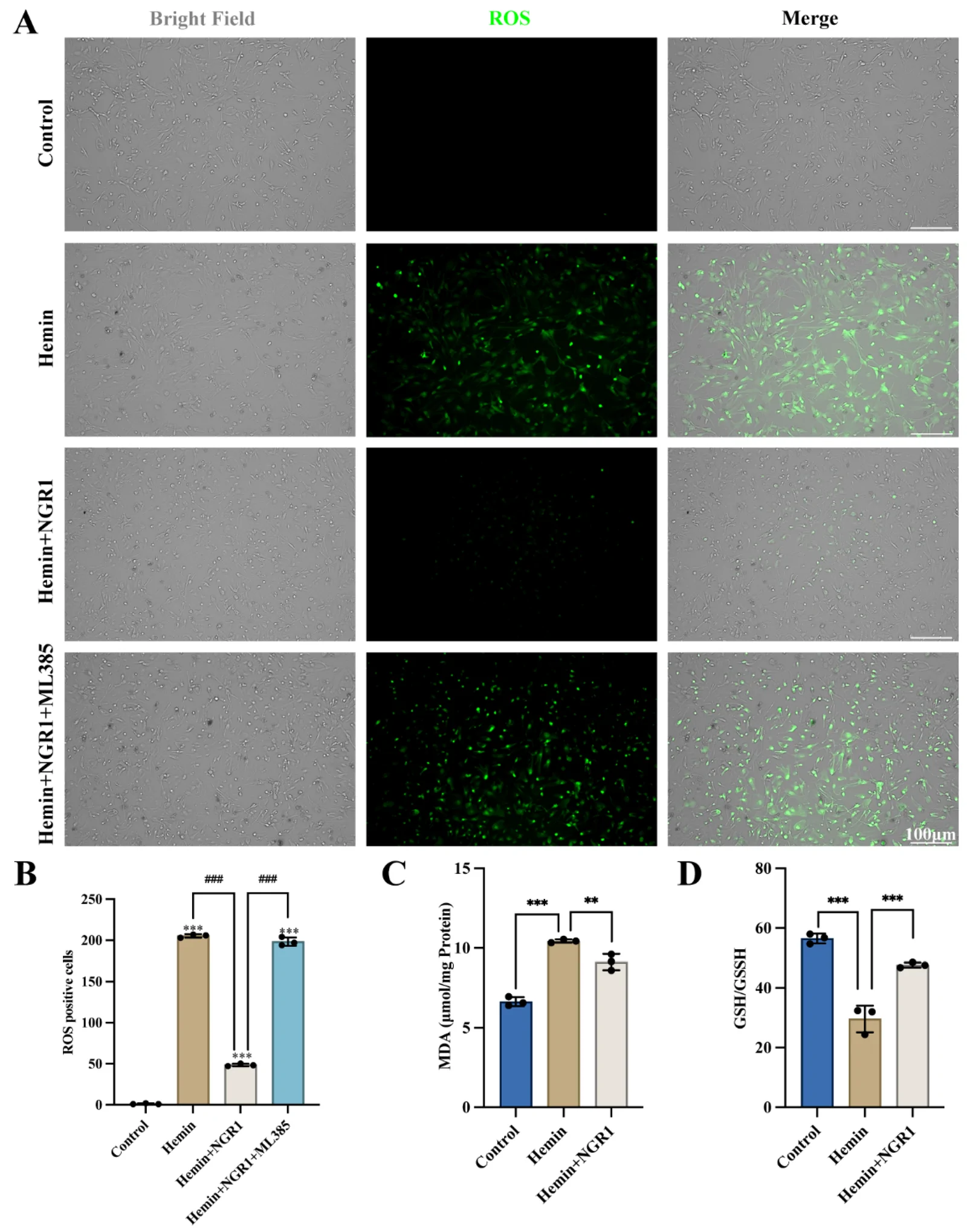
NGR1 ameliorated oxidative injury in hemin-treated astrocytes by suppressing ROS level and MDA level, raising GSH/GSSH ratio. (**A**) Representative fluorescence imaging of ROS marked by DCF (green) in astrocytes pre-incubated with 25μM NGR1 for 24 h followed by 30μM hemin treatment for 24 h. Astrocytes were treated with 10μM Nrf-2/HO-1 inhibitor ML385 for 2 h before NGR1 incubation in hemin + NGR1 + ML385 group. Scale bar = 100 μm. (**B**) The number of ROS-positive cells. n = 3 biologically independent astrocyte cultures. One-way ANOVA followed by Dunnett’s test. *** *p* < 0.001 vs. control group; ### *p* < 0.001, vs. hemin + NGR1 group. (**C**,**D**) Assessment of MDA level (**C**) and GSH/GSSH ratio (**D**) in astrocytes incubated with 25 μM NGR1 for 24 h followed by 30 μM hemin treatment for 24 h. n = 3 biologically independent astrocyte cultures. One-way ANOVA followed by Dunnett’s test. ** *p* < 0.01, *** *p* < 0.001 vs. hemin group. All data are presented as mean ± SD.

To explore the effect and underlying mechanism of NGR1 in oxidative damage, redox states were assessed. Fluorescence imaging of astrocytes labeled with DCF showed that 25 μM NGR1 treatment evidently suppressed hemin-induced ROS levels, while they were reversed by 10 μM ML385 (Figure 6A,B). NGR1 treatment reduced MDA levels in astrocytes (Figure 6C), inhibiting hemin-induced lipid peroxidation. Moreover, NGR1 treatment significantly increased the GSH/GSSG ratio in astrocytes (Figure 6D), suppressing hemin-induced depletion of cellular GSH.

### 3.7. LCN2 Knockdown Promoted HO-1 Expression and Suppressed ROS Level in Astrocytes Treated with Hemin

**Figure 7:**
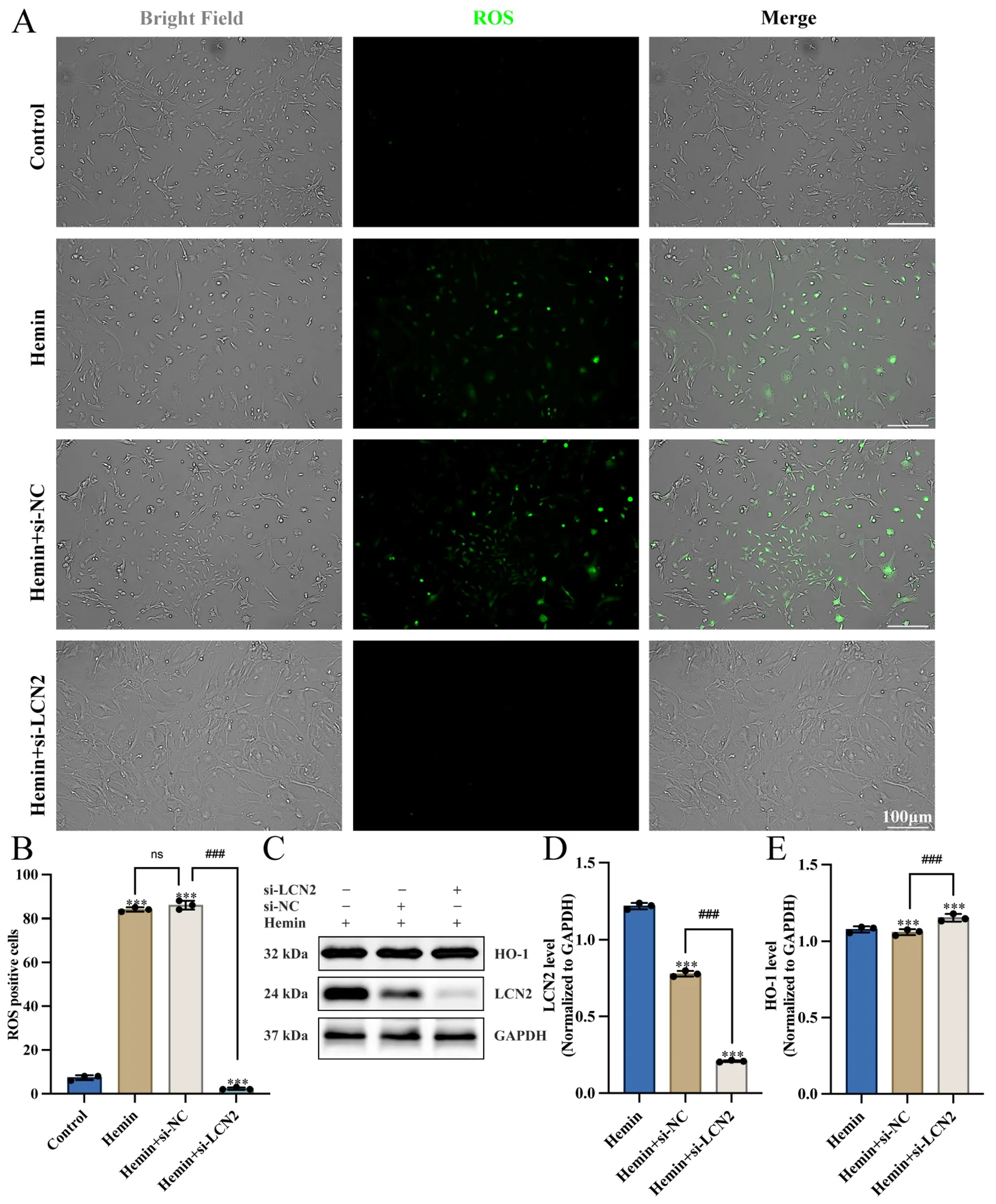
LCN2 knockdown promoted HO-1 expression and suppressed ROS level in astrocytes treated with hemin. (**A**) Representative fluorescence imaging of ROS marked by DCF (green) in astrocytes treated with 30 μM hemin for 24 h. Astrocytes were transfected with 50 nM si-LCN2 for 24 h to knock down LCN2 expression. Scale bar = 100 μm. (**B**) The number of ROS-positive cells. n = 3 biologically independent astrocyte cultures. One-way ANOVA followed by Dunnett’s test. *** *p* < 0.001 vs. control group; ### *p* < 0.001, vs. hemin + si-NC group. ns means no significance. (**C**) Representative immunoblots of LCN2 and HO-1 levels in astrocytes treated with 30 μM hemin for 24 h. Astrocytes were transfected with 50 nM si-LCN2 for 24 h to knock down LCN2 expression. (**D**,**E**) Quantification of LCN2 (**D**) and HO-1 (**E**) protein levels. n = 3 biologically independent astrocyte cultures. One-way ANOVA followed by Dunnett’s test. *** *p* < 0.001 vs. control group; ### *p* < 0.001, vs. hemin + si-NC group. All data are presented as mean ± SD.

To explore the role of LCN2 in oxidative damage, LCN2 was knocked down via siRNA transfection in astrocytes, and HO-1 and ROS levels were detected. ROS levels were significantly increased in astrocytes treated with 30 μM hemin compared to the control group. However, LCN2 knockdown significantly diminished ROS levels compared to the negative control group (Figure 7A,B). Furthermore, si-LCN2 transfection efficiently knocked down LCN2 expression and promoted HO-1 expression compared to the negative control group (Figure 7C–E). These results indicate that LCN2 is associated with oxidative damage, and LCN2 knockdown promotes HO-1 expression and inhibits ROS generation in hemin-treated astrocytes.

### 3.8. HO-1 Knockdown Reversed NGR1-Mediated Suppression of LCN2 and Promoted GFAP Expression in Hemin-Treated Astrocytes

**Figure 8:**
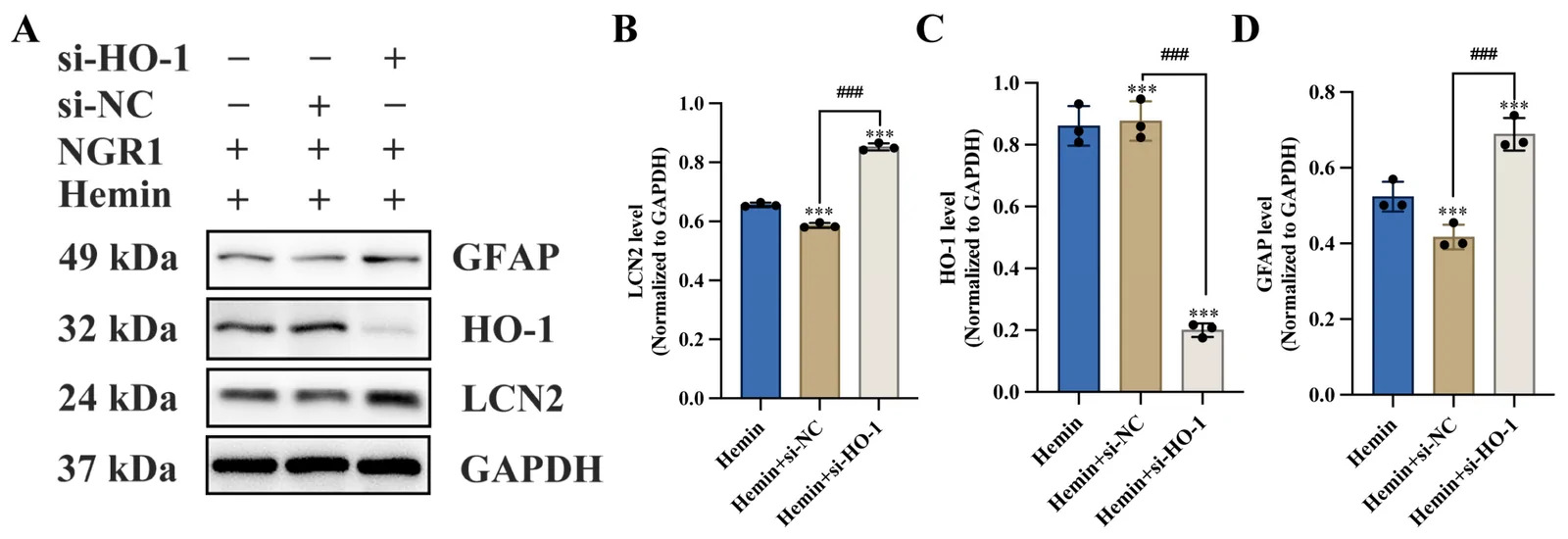
HO-1 knockdown promoted LCN2 and GFAP expression reversed NGR1’s effect in astrocytes treated with hemin. (**A**) Representative immunoblots of LCN2, HO-1 and GFAP levels in astrocytes incubated with 25 μM NGR1 for 24 h followed by 30 μM hemin treatment for 24 h. Astrocytes were transfected with 50 nM si-HO-1 for 24 h to knock down HO-1 expression. (**B**–**D**) Quantification of LCN2 (**B**), HO-1 (**C**) and GFAP (**D**) protein levels. N = 3 biologically independent astrocyte cultures. One-way ANOVA followed by Dunnett’s test. *** *p* < 0.001 vs. hemin group; ### *p* < 0.001, vs. hemin + si-NC group. All data are presented as mean ± SD.

To explore the role of HO-1 in NGR1-mediated protective effects, HO-1 was knocked down via siRNA transfection in astrocytes, and LCN2 and GFAP levels were detected. HO-1 knockdown significantly upregulated LCN2 protein levels in astrocytes treated with 25 μM NGR1 for 24 h followed by 30 μM hemin, compared to the negative control group, thereby reversing NGR1-mediated LCN2 suppression (Figure 8A,B). Moreover, Western blot analysis showed that GFAP protein levels were significantly increased after HO-1 knockdown compared to the negative control group (Figure 8A,D). These results indicate that HO-1 is an important modulator in regulating LCN2.

## 4. Discussion

ICH is a severe type of stroke associated with high global mortality and morbidity, yet an effective therapy strategy is still in urgent demand. In this study, we employed a collagenase-induced mouse model of ICH, established by injecting 0.075 U of collagenase IV dissolved in 0.4 μL of PBS into the striatum to induce cerebral hematoma [34]. Consistent with previous reports, we observed that LCN2 and HO-1 were upregulated in the ICH mouse brain, with both reaching a high level around 3 days after ICH [35]. LCN2 has been reported to be highly expressed in ICH patients and is considered a critical mediator of neuroinflammation and brain injury [36]. In the brain, LCN2 is primarily produced by astrocytes; notably, no co-localization of LCN2 with microglia or neurons was detected following traumatic brain injury [23]. Previous studies have demonstrated that intraventricular injection of recombinant LCN2 exacerbates brain injury, whereas LCN2 knockdown exerts protective effects [37]. Former research has demonstrated that LCN2 is necessary for classical astrocyte activation, and no classically activated astrocytes are detected in LCN2 knockout MCAO mice which leading to better neurological function and smaller infarct volumes [38]. LCN2 has also been shown to mediate oxidative stress and promote neurodegeneration in obesity models [39]. As a siderophore-binding protein, LCN2 is involved in cellular iron transport, innate immune responses, and the regulation of neuroinflammation and neurodegeneration [40]. Iron accumulation contributes to pro-inflammatory processes, disrupts mitochondrial respiration, stimulates oxidative stress, and induces ferroptosis [41]. LCN2 expression was upregulated in astrocytes in the brain of ICH mouse, while iron chelators inhibited LCN2 production in primary astrocytes [42]. Extensive research indicates that LCN2 plays a pivotal role in promoting brain injury and iron toxicity in ICH [43]. While low levels of LCN2 facilitate the transport of physiological iron, pathological increases in LCN2 lead to enhanced binding of iron-siderophore complexes, contributing to iron accumulation [44,45,46]. Elevated LCN2 suppresses autophagy and promotes ferroptosis, thereby aggravating brain damage [47,48,49]. Hemin, a porphyrin complex derived from erythrocytes, induces HO-1 expression, which subsequently cleaves hemin to generate carbon monoxide (CO), iron, and biliverdin [50,51]. Iron released from the hematoma induced LCN2 upregulation in ICH, while it was inhibited by the iron chelator deferoxamine [52]. HO-1 is regarded as a critical driver of iron-mediated oxidative pathology [53]. However, past research has revealed that HO-1 exhibits anti-inflammatory and antioxidant properties [54]. Previous studies reported that HO-1 is stimulated as an oxidative stress response, with transcript levels upregulated within 24 h and protein levels peaking at 3 days post-cortical contusion injury (CCI) [35]. Earlier observations noted that HO-1 upregulation occurs earlier in astrocytes than in neurons [55]. Moreover, HO-1 plays a pivotal role in mitigating oxidative damage, and astrocyte-selective HO-1 overexpression provides robust neuroprotection and improves outcomes after ICH [10]. Consistent with previous findings, our results showed that LCN2 was upregulated 24 h after ICH and remained at high levels through day 3, preceding the peak of HO-1 expression [35]. Based on this evidence, we hypothesized that inhibiting LCN2 or promoting HO-1 expression could alleviate brain damage following ICH.

Microglia were traditionally considered the primary immune cells in the brain, participating in phagocytosis and pro-inflammatory cytokine production [56]. However, astrocytes have increasingly been recognized as essential players in neuronal inflammation and oxidative damage [16]. Hemin is a key contributor to secondary brain damage after ICH, facilitating lipid peroxidation by intercalating into cell membranes, exacerbating the depletion of cellular glutathione (GSH) stores, and escalating hydroxyl radical production via the Fenton reaction [51]. In this research, astrocytes were treated with 30μM hemin to mimic the ICH-induced brain injury. In accordance with previous reports, our findings indicated that hemin activated astrocytes and significantly induced LCN2 expression [57]. LCN2 plays a crucial role in determining the phenotype of activated astrocytes [22]. We found that LCN2 promoted reactive oxygen species (ROS) generation in pro-inflammatory reactive astrocytes, whereas LCN2 knockdown suppressed ROS levels. This is consistent with recent reports showing that LCN2 mediates ROS generation in bone marrow-derived dendritic cells [26]. Aligned with an earlier report that LCN2 knockdown promoted HO-1 expression in Bend.3 cells [37], we observed that LCN2 knockdown resulted in the upregulation of HO-1. We speculate that oxidative damage in LCN2-mediated pro-inflammatory reactive astrocytes is closely associated with HO-1 activity.

NGR1 has been reported to possess antioxidant properties and plays an important role in the treatment of neurological disorders [27]. To explore the effects of NGR1 on ICH and its underlying mechanisms, we administered NGR1 to both in vivo and in vitro ICH models. Earlier studies have revealed that NGR1 prevents neurological deficits and mitigates brain edema in mice with subarachnoid hemorrhage [58]. NGR1 also proved to induce neuroprotective and anti-inflammatory effects in MCAO-induced cerebral ischemia [59,60,61]. Consistent with previous studies, our results showed that 20 mg/kg NGR1 reduced hematoma volume and brain water content at 3 days post-ICH and improved neurobehavioral performance. While earlier studies indicated that hemin causes lipid peroxidation and depletes cellular GSH [9,51,62], we found that NGR1 treatment lowered malondialdehyde (MDA) levels and increased the GSH/GSSH ratio. NGR1 has been shown to promote HO-1 expression and suppress ROS levels in HK-2 cells, thereby attenuating oxidative damage [29]. Our results demonstrated that NGR1 significantly reduced ROS levels in astrocytes, which agrees with previous reports that NGR1 suppresses ROS generation and promotes ROS clearance in PC12 cells [30]. Previous research has shown that NGR1 attenuates oxidative stress, neuronal apoptosis, and inflammation after spinal cord injury by activating the Nrf-2/HO-1 signaling pathway, which was partially reversed by the HO-1 inhibitor ML385 [63]. Consistent with previous findings, we found that NGR1 inhibited LCN2 levels and promoted HO-1 levels in astrocytes, whereas ML385 reversed the NGR1-mediated suppression of ROS levels and LCN2 expression. Furthermore, our results demonstrated that HO-1 knockdown elevated LCN2 levels in astrocytes, reversing the protective effect of NGR1. Abundant research has demonstrated that NGR1 ameliorates inflammation by suppressing the NF-κB signaling pathway [27,64,65]. NF-κB is an essential regulator of LCN2 transcription, which is predominant in glial cells in the brain [40,66]. Moreover, previous studies have shown that the Nrf-2/HO-1 pathway can downregulate the NF-κB pathway, promoting neuroprotection by inhibiting oxidative stress and neuroinflammation [67,68]. Additionally, spinal glial activation was inhibited by downregulating the Nrf2/HO-1/NF-κB pathway in an inflammatory pain mouse model [47]. Our results indicate that NGR1 suppresses LCN2 expression by upregulating HO-1, inhibits the polarization of astrocytes toward a pro-inflammatory phenotype, and attenuates oxidative damage and inflammation after ICH.

There are several limitations to this study. The ICH model was induced in C57BL/6 mice aged 8 weeks, whereas human ICH is more prevalent in older populations. Therefore, further studies in aged mice would be valuable for exploring age-related neuroinflammation. In our research, the knockdown of HO-1 or LCN2 in astrocytes revealed that NGR1 protects against brain injury by suppressing LCN2, which may be associated with the crosstalk between the Nrf-2/HO-1 and NF-κB pathways, as suggested by previous reports. Further investigation into the effect of LCN2 overexpression on HO-1 and related pathways in vivo and in vitro might help clarify the mechanism more precisely. Moreover, while we investigated oxidative injury induced by hemin, LCN2 is also associated with cellular iron transport. Although abundant research demonstrates that LCN2 is related to poor outcomes in traumatic diseases, further exploration of whether NGR1 suppresses brain damage via inhibiting LCN2-associated iron toxicity would be meaningful.

## 5. Conclusions

In this research, we investigated whether NGR1 could alleviate brain injury induced by ICH. The *in vivo* study showed that 20 mg/kg NGR1 administration attenuated cerebral edema, reduced the hematoma volume and improved the neurobehavior outcome by inhibiting LCN2 upregulation and inducing HO-1 expression after ICH. The *in vitro* study revealed that 25 μM NGR1 inhibited LCN2, ROS and MDA levels and upregulated the HO-1 level and GSH/GSSH ratio in hemin-treated astrocytes. Furthermore, LCN2 knockdown promoted HO-1 expression and suppressed ROS generation. Moreover, HO-1 knockdown or Nrf-2/HO-1 inhibitor ML385 treatment partially reversed the protective effect of NGR1 and promoted the expression of LCN2 and GFAP.

In conclusion, this study illustrates that NGR1 alleviates ICH-mediated oxidative damage and inflammation via the HO-1/LCN2 axis, indicating that NGR1 may be a promising therapeutic strategy for ICH.

## Data Availability

All data generated or analyzed during this study are included in this article.

## Supplementary Materials

The following supporting information can be downloaded at https://www.mdpi.com/article/10.3390/brainsci16090958/s1, Figure S1: Schematic diagram stereotactic coordinates and brain regions observed [69].
