# Supplementary material for: Notoginsenoside R1 Attenuates Oxidative Damage After Intracerebral Hemorrhage by Inhibiting LCN2 and Promoting HO-1 in Astrocytes
Source: Brain Sci. 2026 Sep 10;16(9):958. doi: 10.3390/brainsci16090958 (PMC13604683; doi:10.3390/brainsci16090958)
Supplement: Supplementary file 1 [file brainsci-16-00958-s001.zip › brainsci-4022695-supplementary.pdf]

Supplementary Materials:

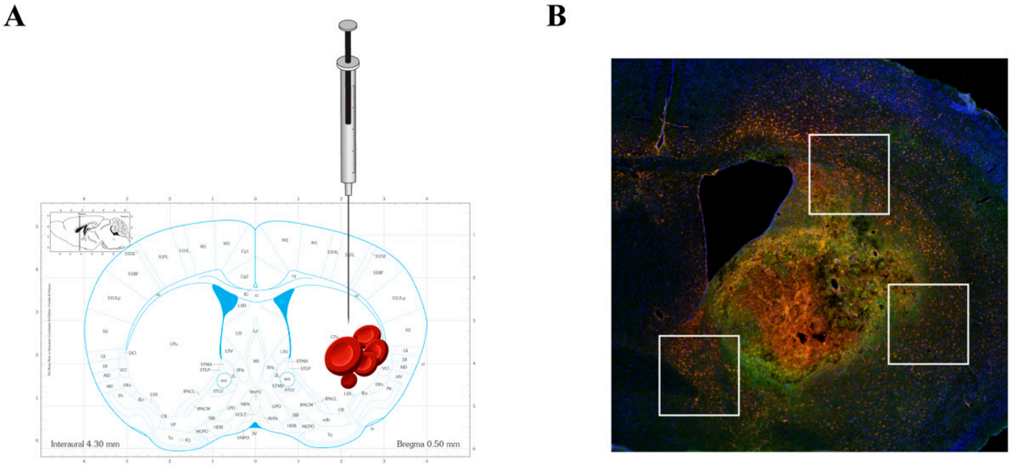

Supplementary Figure S1. Schematic diagram stereotactic coordinates and brain regions observed. (A) Schematic diagram of collagenase injected into the striatum（3 mm deep, 0.5 mm anterior and 2.2 mm lateral to the bregma）to induce the hematoma in the brain. CPu, caudate putamen (striatum). The stereotaxic coordinates was in reference to The Mouse Brain in Stereotaxic Coordinates (Third Edition) [69]. (B) Schematic diagram of brain regions used for the immunofluorescence analysis. The prelesion area was the region around the hematoma, 3 non-overlapping fields in coronal slices for each animal were randomly chosen for imaging.

Reference

69. Keith, B.J.; Fraklin, G.P. *The Mouse Brain in Stereotaxic Coordinates*, 3rd ed.; Academic Press: Cambridge, MA, USA, 2007.
